# Depression in adolescence and young adulthood: the difficulty to integrate motivational/emotional systems

**DOI:** 10.3389/fpsyg.2024.1391664

**Published:** 2025-01-06

**Authors:** Teodosio Giacolini, Antonio Alcaro, David Conversi, Lorenzo Tarsitani

**Affiliations:** ^1^Department of Human Neuroscience, Sapienza University of Rome, Rome, Italy; ^2^Department of Psychology, Sapienza University of Rome, Rome, Italy

**Keywords:** adolescence, depression, dominance system, motivational-emotional systems, hormones, neoteny, young adulthood

## Abstract

Depression is presented as a multi-factorial bio-psycho-social expression that has evolved primarily as an effect of stressors related to the motivational/emotional systems that regulate the *BrainMind* in our relationship with conspecifics. These stressors may be caused by two sources of threat, firstly, the loss of bonding with the caregiver and later with a partner and/or group which relates to the SEPARATION (PANIC/GRIEF) system, secondly, social defeat as an expression of the social competition and social dominance. The sexual maturity drives the individual to social competition and social dominance, even if the latter often occurs before sexual maturity, e.g., chickens, dogs, non-human primates, and humans. Depression is an evolutionarily conserved mechanism in mammals to terminate both separation anxiety, so as to protect the vulnerable social brain from the consequences of prolonged separation anxiety, and the stress of social competition when social defeat is predictable. Adolescence and Young adulthood are particularly susceptible to these two types of threat because of human developmental characteristics that are summarized by the term *neoteny*. This refers to the slowing down of growth and development, resulting in both a prolonged period of dependence on a caring/protective adult and the persistence of juvenile characteristics throughout life. Therefore, *neoteny* makes the transition from childhood to sexual maturity more dramatic, making the integration of the SEPARATION (PANIC/GRIEF) system with the dynamics of social competition and dominance more stressful and a source of depression. Stress is an expression of the *HPA-Hypothalamic-Pituitary-Adrenal* axis that articulates with other systems, mainly the *autonomic nervous system* and the *immune-inflammatory system*. The latter is believed to be one of the most significant components in the dynamics of depressive processes, connected to the prodromes of its activation in childhood, under the pressure of environmental and relational stressors which can lead to *learned helplessness*. The recurrence of stressors makes it easier for the *immune-inflammatory system* to be activated in later life, which could make a significant contribution to the establishment of a depressive disease. The possible contribution of children's identification processes with their parents' depressive personalities through observational learning is considered.

## Introduction

Depression when considered from a comparative psychobiology point of view can be traced back to some bio-psycho-social function models (Watt and Panksepp, 2009, p. 42 Appendix). The most significant is the separation distress model (Bowlby, 1980), about which Panksepp (2010, p. 540) writes: “the separation-distress/GRIEF “protest” gateway to depression.” In this model depression is as an evolutionarily conserved mammalian mechanism that terminates separation distress, so as to protect the vulnerable social brain from the consequences of prolonged separation anxiety. This mechanism is used later in life also to terminate social anxiety when defeat in social competition is predictable (Watt and Panksepp, 2009; Watt, 2023).

In mammals, and especially in the human species, reactions to the threats of separation and social competition are under the control of two motivational/emotional systems, namely, the SEPARATION (PANIC/GRIEF) system (Panksepp, 1998) and the Dominance system (Van der Westhuizen, 2013). The former has priority during childhood while the latter especially after sexual maturity (Bos et al., 2007; Holder and Blaustein, 2014), when: “the separation distress system seems to exhibit…a gradual decline beginning from puberty” (Panksepp, 1998, p. 56). The maturation of sex hormones, in close interaction with the development of various brain areas, drives adolescents and young adults toward peers as a source not only of potential reward but also of social defeat during social competition under Dominance system pressures. The SEPARATION (PANIC/GRIEF) system, however, continues to function. The simultaneous existence of these two systems involves processes of integration between both of them especially for the effect of *neoteny* (Gould, 1977). The latter refers to the phenomenal slowing down of growth and development, that results in both an extension of the period of dependence on a protective adult and the persistence of juvenile characteristics throughout life (Lorenz, 1971). *Neoteny* has extended and made more significant and complex the dynamics of learning through exposure to primary reference models, namely, caregivers. This learning becomes the subject's procedural and regulatory heritage through the provision to mimesis, a function present from birth (Bandura, 1977; Marshall and Meltzoff, 2014; Meltzoff and Moore, 1977) and the expression of specific areas of the brain called *Mirror neurons* (Gallese et al., 1996; Rizzolatti et al., 1996). While these features have probably played a major role in determining the human species' extraordinary ability to live in complex and cooperative social groups (Woodward and Gerson, 2014; Tomasello, 2014), they have also contributed to making it vulnerable to mental illness, particularly depression. We can assume that neural mirror systems are involved in transgenerational dynamics (Ferrari et al., 2013), through which traits related to the regulation or dysregulation of motivational/emotional systems, such as depression, are transmitted from one generation to the next.

The synergy between learned vulnerability, biopsychic responses to specific relational stressors at different stages of development, and the difficult to integrate SEPARATION (PANIC/GRIEF) and DOMINANCE systems through the effect of neoteny, particularly in adolescence and young adulthood, promotes depressive malaise which is a source of stress and involves activation of the HPA stress axis and the immune-inflammatory system (Watt, 2023).

For the purposes of this article we will use the term “Dominance system” even if there is currently no consensus among scientists as to whether it is a primary emotional system or an expression of the interaction of other primary emotional systems (Panksepp and Biven, 2012; van der Westhuizen and Solms, 2015). Panksepp and Biven (2012) that social dominance is a secondary learned system, and thus not a phylogenetic system in the sense of a primary emotion. The Dominance system is fully functioning with the advent of sexual maturity, even if social dominance often occurs before sexual maturity, for example, in chickens, dogs, non-human primates, and humans. During childhood, hierarchies of peer dominance are formed (Hawley, 1999, 2007; Pellegrini et al., 2007, 2011), in which children can feel the emotions connected to being dominant or submissive.

Depression from a psychobiological perspective has been extensively investigated in animal models. These preclinical models are designed to produce situations whose effects are interpreted as analogous to the symptoms of depression in humans. These include situations that produce not only separation distress (Panksepp, 1998; Panksepp and Biven, 2012; Watt and Panksepp, 2009) but also social defeat (Heshmati et al., 2020; Rodriguez et al., 2024; Yoshida et al., 2021), the loss of status (D'Aquila et al., 1995; Fan et al., 2023; Harris and Padilla-Coreano, 2023), *learned helplessness* (Maier and Seligman, 2016; Seligman and Maier, 1967) and other chronic stress situations (Gencturk and Unal, 2024; Henn and Vollmayr, 2005; Pryce et al., 2011). However, the results of all these preclinical models raise the question of whether such animal phenotypes are genuinely analogous to depression in humans (Flandreau and Risbrough, 2024; Gencturk and Unal, 2024). Indeed, while rodents are widely used in laboratories for easy breeding, such animals have species-specific characteristics that are relatively lacking authentic separation distress (Panksepp and Biven, 2012, p. 321–22).

Moreover, these preclinical models lack the relational and experiential dimensions essential to human depression pathogenesis.

## Neoteny in the human species

The study of the evolution and differentiation of species has highlighted the dynamics of development as a strategic element. This is highlighted by the phenomenon of *heterochrony* (McKinney and McNamara, 1991), which is a “change in the timing or rate of developmental events, relative to the same events in the ancestor” (Alberch et al., 1979; McKinney and McNamara, 1991; McNamara, 2012) resulting in changes in the size and shape of morphology and behavior (Gould, 1977; McKinney, 1999). In humans*, heterochrony* was identified as *neoteny*. This latter is, therefore, a “*paedomorphosis produced by the retardation of somatic development*” (Gould, 1977, p. 483). This slowing down of growth in size and somatic form, coupled with the retention of infantile or juvenile behavioral, motivational and emotional traits into adulthood, has favored in the human species exceptional cerebral development and learning ability, together with the sexual maturation of the individual which occurs while still in a pre-adult stage of development (Bogin, 1999; Bufill et al., 2011).

Darwin had already highlighted development as being central to the evolution of species in the *Origin of Species*, in the chapter “Development and Embryology,” stating that: “*this is one of the most important subjects in the whole round of natural history*” (Darwin, 1959 6th edition 1878, p. 386; McNamara, 2012). Interest in development and *heterochrony* declined dramatically until it disappeared from the study of biological disciplines (McNamara, 2012), along with interest in evolutionism, with the rise of research in genetics in the early 1900s (Hofer, 2014). A revival of interest in the biology of developmental processes occurred in the 1970s with the publication of S. Gould's textbook *Ontogeny and Phylogeny* (Gould, 1977). This book rekindled interest in the centrality of developmental processes in the evolution of species and specifically in the human species. It contributed significantly to the emergence of the so-called EVO-DEVO (*evolutionary developmental biology)* discipline (Hall, 2003). This discipline has taken up the study of changes in the developmental mechanisms of phenotypic aspects through *life history* events (Hochberg, 2009; Del Giudice, 2009), mainly related to the dimension of organic functioning and neural plasticity (Hochberg, 2011), but also to the behavioral dimension (Lorenz, 1971), although the latter has been much less studied.

## Neoteny and motivational systems

*Neoteny* prolongs the period of offspring dependence on caregivers, which is beneficial for brain development and learning. Both in humans and in other mammals, as shown in particular by research on rats and monkeys (Tottenham, 2020). At the same time, it optimizes the use of energy resources (*optimal energy allocation*) for the goals of body growth and reproduction in the life-history strategy (Del Giudice, 2009; Ellison, 2016; Hochberg, 2011). In the human species, the stages of growth (i.e., physical and biological changes) and development (i.e., functional and behavioral changes) have seen the appearance of childhood and adolescence stages (Bogin and Smith, 1996), that do not exist in other mammals. In most of the latter, there are only two developmental stages, infancy and adulthood, the transition from one to the other is quite sudden, and sexual maturity occurs while growth rates are declining (Bogin and Smith, 1996).

The emergence in the human species of the two stages of childhood and adolescence has meant that the slowing down of growth, with the need for longer dependence on the caregiver, has not affected the latter's reproductive rate, avoiding the prolongation of the lactation period (Bogin and Smith, 1996; Key, 2000). This problem was “solved” with the appearance of the childhood stage (3-6aa) soon after weaning. Childhood makes it possible to reduce the primary dependence of the breastfeeding period, although it is still characterized by considerable dependence on the caregiver. In fact, this is a period in which the immaturity of the dentition and the digestive tract requires food to be prepared by the adult caregiver that is easily digestible, but also highly energetic due to the demand for substances necessary for growth, especially of the brain, which has its highest growth rate during this period (Roxas, 2021; Stiles and Jernigan, 2010). Childhood is followed by the *juvenile* age, 6–11 years, characterized by relative autonomy from caregivers. This is followed by puberty, brought about by the maturation of the gonads, the opening phase of the adolescent period in the human species, characterized by a species-specific peak in skeletal and muscular growth, as well as continued brain development and intense learning (Jaworska and MacQueen, 2015; Reddy et al., 2022). In the last two or three decades, adolescence has been joined by the identification of a further developmental period between 18 and 25 years of age, called young adulthood (Arnett, 2010; Hochberg and Konner, 2020; Higley, 2019). This period has specific biological and socio-cultural characteristics.

The neotenic slowdown, “…we are neotenous creatures who benefit from a much longer childhood than other species.” (Panksepp, 1998, p. 287), required the integration of different motivational/emotional systems evolved at different times in the evolution of the human species (Panksepp and Biven, 2012). The dynamics of this integration should be identified as one of the causes of the emergence of psychopathology, which begin on a large scale in adolescence (Del Giudice and Ellis, 2016; Patel et al., 2021). The integration of different motivational/emotional systems is an event that characterizes what is known as the *developmental cascade* (Masten and Cicchetti, 2010; Jones et al., 2016; Lin et al., 2020). The latter refers to processes by which the function and dynamics of one system affect other systems or levels of function, shaping the course of ontogeny and epigenesis (Masten and Cicchetti, 2010). This is due to the cumulative consequences of the many reciprocal interactions and transactions that occur in the development and dynamics of motivational/emotional systems.

The stages of growth and development are interconnected with the evolutionary and developmental dynamics of the motivational/emotional systems that support interaction and adaptive response to the surrounding world, and particularly the relational world. In the human species, the relational dimension has become the new Environment of Evolutionary Adaptedness (EEA) (Bennett, 2018; Bowlby, 1969). It drives the functional integration of motivational/emotional systems (Panksepp and Watt, 2011) that have evolved at the different periods of phylogeny (MacLean, 1990).

Motivational/emotional systems have been studied in recent decades by Affective Neuroscience (Panksepp, 1998), which has identified and/or systematized studies of the systems involved in the regulation of human interactions. These systems are, in a hypothetical evolutionary order of appearance, the following: the SEEKING system, moves the animal to look for resources; the RAGE system, moves the animal to defense against predators with counter-aggression or defend resources; the FEAR system, moves the animal to avoid danger; the LUST system, identifies potential mates and reproduce; the CARE system, move mammals to care for offspring; the SEPARATION (PANIC/GRIEF) system, promotes social bonding; the PLAY system, moves mammals to physically play to learn and build social bonds (Panksepp, 1998). There is another motivational/emotional system called the Dominance system, that is not a primary emotional system in the classical sense (Panksepp, 1998; Karterud and Kongerslev, 2019; van der Westhuizen and Solms, 2015), even if, it is evolutionarily archaic (MacLean, 1990, p. 99–113; Toronchuk and Ellis, 2013). This system is formed by the complementary interaction of the RAGE and FEAR systems and it is present in all vertebrates and regulates the interactions between sexually mature conspecifics for access to food and sexual resources.

SEEKING, LUST, FEAR, and RAGE are the earliest phylogenetically systems, which all vertebrates possess, whereas CARE and SEPARATION (PANIC/GRIEF) system are present both in mammals and in some bird species. Mammals exhibit social rough and tumble PLAY while birds may exhibit object play. The CARE and SEPARATION (PANIC/GRIEF) systems regulate the interaction between offspring and caregivers, while PLAY contributes to cooperative group life (Panksepp and Biven, 2012). Specifically, CARE is the parental care system and SEPARATION (PANIC/GRIEF) is an alarm system that favors the attachment bond. CARE and SEPARATION (PANIC/GRIEF) systems primarily regulate interactions between offspring and caregivers involving *opioids, oxytocin* and *prolactin* (Burkett and Young, 2012; Panksepp and Biven, 2012). These two systems are the regulators of wellbeing when the functional relationship between offspring and caregiver is active, or of discomfort when it is prevented for some reason. The first malaise for a mammal is the experience of separation from the caregiver with the activation of the SEPARATION (PANIC/GRIEF) system, when the sexual system is not yet developed (Burkett and Young, 2012; Panksepp and Biven, 2012). The experience of separation distress involves both reduced *opioid* release and the involvement of the *extended amygdala*. The latter is a network consisting of interactions between the *medial* and *lateral nuclei* of the *amygdala* (CeA, MeA), the *bed nucleus stria terminus-BNST*, and the *nucleus accumbens shell* (NAcSh) (Alheid, 2003; Giardino et al., 2018; Klumpers et al., 2018). The *BNST* is sensitive to the detection of threat signals associated with maternal separation, resulting in anxiety, dysphoria and anhedonia. It promotes the expression of malaise through the behaviors of anxious recall to caregivers and withdrawal when the former does not lead to the reestablishment of a social bond (Halladay and Herron, 2022). These data must be read with the *caveat* that it mostly uses studies on the separation of rat pups from their mothers, which is controversial because of a greatly diminished SEPARATION (PANIC/GRIEF) system due to being bred to live in isolated laboratory conditions (Panksepp and Biven, 2012, p. 321–322).

The study of motivational/emotional systems (Panksepp, 1998) has documented how the subcortical component of the brain is a phylogenetic inheritance that is the source of instinctual and emotional behaviors that shape cortical functioning and regulate relational life (Panksepp, 2011). Emotions and the neural structures that support them have traditionally been considered evolutionarily conservative, particularly those associated with the *limbic system* (Barger et al., 2014). The evolution of the human species has favored an extraordinary expansion of the brain, and in particular of the cortex, which has been the subject of intense research; less studied is the parallel evolution of the *limbic system* and *subcortical* components involved in emotional systems and, specifically, in the regulation of social interactions (Barger et al., 2014). Of particular importance in humans is the increased volume of the (lateral) *amygdala* compared to other primates (Barger et al., 2007). Other areas of the *limbic system* (*hippocampus and orbital frontal cortex*) are involved in the evaluation of social interactions (Barger et al., 2014).

## Neoteny and the FEAR system

The previous section considered the reaction to a threat, a reaction of both anxiety and protective withdrawal, in which *the extended amygdala* plays a central role in regulating these reactions. *Neoteny* in the human species is a particularly important perspective when considering the development of the *amygdala*'s connections with the *medial prefrontal cortex* (mPFC) (Liu et al., 2020; Marek et al., 2013; Zimmermann et al., 2019), through which the regulation of emotions occurs. This is influenced by early stress responses associated with environmental threats. The connectivity between the *amygdala*, which is rich in stress hormone (*cortiso*l) receptors especially in infancy, and the *medial prefrontal cortex* (mPFC), which is functional in regulating the former, is present from the first year of life and undergoes continuous changes in the following years (Goodman et al., 2022; Koppensteiner et al., 2014; Meisner et al., 2022; Tottenham, 2020; Tottenham and Gabard-Durnam, 2017). This initial connection will influence an adult's later connection and thus affects how an adult will regulate emotions. The connections between the *amygdala* and *mPFC* are, therefore, still developing in childhood, hence the particular reactivity to threatening stimuli in infants and primarily the alarm at the perceived separation of the caregiver (Tottenham, 2015). The *amygdala*-*mPFC system* slowly develops in humans due to *neoteny*, in contrast to other species. During infancy, this system is vicariously influenced by the caregiver relationship, whose presence has the function of modulating *amygdala* activation to threatening stimuli and reducing its reactivity (Norman et al., 2015; Tottenham, 2020). The vicarious modulatory function of the caregiver on the *amygdala* continues until sexual development, after which the development of the *amygdala-mPFC system* in adolescence renders this vicarious modulation less effective (Gee et al., 2014; Hare et al., 2008). However, adolescents show less risk-taking behavior when their mothers are present than when they are alone in a particularly stressful situation, reducing *amygdala* activation (Telzer et al., 2015).

## FEAR system and *Social buffering*

The developmental characteristics of the *amygdala-mPFC* connection are thus one of the effects of human *neoteny*. In other species, this maturation occurs at an early age, allowing offspring to discriminate and cope with environmental stressors. During infancy, attachment means that proximity to the caregiver allows exploration of the physical and relational world, through what is known as *Social buffering* (Kikusui et al., 2006; Sanchez et al., 2015), without the need for the *amygdala-mPFC* system to be fully developed. *Social buffering*, therefore, is the phenomenon by which the presence of a familiar individual reduces or even eliminates stress and fear induced responses (Sanchez et al., 2015). *Social referencing*, which is an infant's tendency to seek information from a caregiver in order to regulate behavior toward an ambiguous referent (Ehli et al., 2020), may be further evidence of this phenomenon. Therefore, the presence of a parent acts as a framework/support for the *amygdala-mPFC* circuitry, producing an instantaneous pattern of *amygdala-mPFC* connectivity in children similar to that in adults, which modulates not only the *amygdala* activity (Gee et al., 2014; Tottenham, 2020) but also, simultaneously, the release of *dopamine* in the *basolateral amygdala* (Barr et al., 2009; Ferrara and Opendak, 2023; Opendak et al., 2019). In fact, the effect of *dopamine* release is to increase the response of the *amygdala* by decreasing the inhibitory influence of *prefrontal* inputs and increasing the excitatory influence of threatening stimuli, allowing the formation of fear-related memories. Parental bonding and caregiving styles influence this regulation in developing individuals by modulating responses to fearful stimuli in a manner similar to caregivers (Chen et al., 2020; Tottenham et al., 2019). Furthermore, during childhood, the parental bonding ensures that fearful signals from the caregiver, threats or abuse, do not lead to defensive distancing, but the child still seek closeness, as they learn them to be functional signals so as to stay close to the caregiver, which can result in a high possibility of insecure attachment and risk of psychopathology, particularly mood disorders, in adolescence (Barbaro, 2020; Duprey et al., 2022; Main and Solomon, 1986; Stronach et al., 2011). If the parent is the source of the infant's fear, this places the infant in an insoluble paradox as to whether to turn to the parent for comfort. This is because the parent becomes both a source of the infant's fear and a haven of safety (Main and Hesse, 1990). Exposure to parents who establish insecure attachments, resulting in the learning of such characteristic relationship patterns, may lead to the search for sexual partners with similar characteristics in adolescence and young adulthood, resulting in subsequent experiences characterized by stress and depression (Fraley and Shaver, 2000; Hazan and Shaver, 1987; Umemura et al., 2017).

The presence of the caregiver during childhood with a vicarious function of *amygdala-mPFC* modulation inhibits *amygdala* growth, whereas stress caused by maternal neglect, abuse or depression leads to an increase in *amygdala* volume and its increased reactivity, together with a reduction in the plasticity of *amygdala-mPFC* connections (Granat et al., 2017; Lupien et al., 2011; Heller et al., 2016; Teicher et al., 2016). Notably, the *prefrontal cortex* development lasts until the third decade of life, much longer than in other animal species (Petanjek et al., 2011), as synapses are reorganized from overproduction to elimination. The *neotenic* effect of parent-offspring interaction on the FEAR system keeps the *amygdala-mPFC* circuit inhibited in growth until the pubertal processes of somatic and hormonal growth force the subject to separate from their parents and move toward peers. As regards development of the separation processes of offspring from parents Panksepp and Biven (2012, p. 323) writes: “Young animals are very dependent on it, but with maturation the responsivity of the system diminishes, partly because of the inhibitory effects of sex steroids, which shift animals toward adult form of socio-sexual gratifications.” This relational change leads to a change in the *amygdala-mPFC system*, with the development in the former and an increase in connectivity in the circuit. This change makes the subject better able to navigate between environmental and relational threats (see Tottenham, 2020).

## Neoteny, SEPARATION (PANIC/GRIEF) system and depression

The SEPARATION (PANIC/GRIEF) system, when faced with the occurrence of separation, activates, as mentioned above, an emotional and behavioral reaction called *Protest* (Bowlby, 1969; Watt and Panksepp, 2009) to alert the caregiver, followed, if unsuccessful, by the *Despair* phase, characterized by withdrawal and anhedonia. The state of separation alarm activates the stress axis *hypothalamic–pituitary–adrenal axis (HPA axis)* which, if the alarm situation persists, it removes the trigger for parental search. This leads to the manifestations of withdrawal and anhedonia that are characteristic of depression in the human species (Watt and Panksepp, 2009). The withdrawal of the *Despair* phase is characterized by the depletion of *opioids*, a source of wellbeing when they are present. In the separation distress situation, they are “replaced” by other *opioids, dynorphins*, which induce a state of malaise with a concomitant reduction in *dopamine* release (Watt and Panksepp, 2009). This dynamic is intensified in females by the presence of *estrogen* (Arnsten, 2015). This is associated with females exhibiting greater neoteny than males (Brüne, 2000). The behavioral model of *Despair* (Bowlby, 1969) in neurobiological research is a shutdown mechanism to terminate prolonged separation distress which, if sustained, would be a potentially metabolically exhausting for infant mammal (Watt, 2023; Watt and Panksepp, 2009). In the human species, depression is characterized by dysphoric, anxious, withdrawal, anhedonic states and negative implicit expectations (Kube et al., 2020). This state of depressive malaise has the function of activating in a complementary way the preoccupied approach of the conspecifics, which is particularly evident in parental care (Fernandez-Duque et al., 2009; Panksepp and Biven, 2012). The dynamics of the mammalian parental-infant bonding are more complex in the human species, as suggested by the *fearful ape hypothesis* (Grossmann, 2022, 2023). This theory (Grossmann, 2022, 2023) suggests that in humans, during childhood, the expression of fear caused by new unknown experiences, especially in relationships, and the individual's interest in people who express fear, are the opposite sides of the same coin. This trait has evolved within the *life history* of the human species, which for a long time was characterized by living in small groups, where the care of offspring had to be largely shared with other members of the group, leading to *alloparental care* (Lancaster and Lancaster, 1987; Sear and Mace, 2008). This cooperative breeding is said to have facilitated the evolution of *heightened fearfulness* as an emotional trait (Grossmann, 2022, 2023) by fostering an approach of caring for children by the group to which they belong, while at the same time implementing the cooperative capacity of the human species (Tomasello, 2014). The increased ability to tolerate the proximity of conspecifics, with a reduction in defensive aggression, is associated with both a reduction in *acetylcholine* and, in particular, an increased release of *serotonin, dopamine and oxytocin* (Carter, 2014; Hirter et al., 2021; Raghanti et al., 2008). The *dopaminergic system* via the *ventral striatum*, which has connections to both the *amygdala* and *prefrontal cortex*, is involved in facilitating of reward from the relational dimension (Feldman, 2012). The FEAR system, characterized by both behavioral reactivity, flight and an inhibited approach to new situations, objects and people (Gartstein and Rothbart, 2003; Grossmann, 2022; Kagan and Snidman, 2004), and contributes to the personality structure together with the other motivational/emotional systems mentioned above (Davis et al., 2003; Davis and Panksepp, 2011). Increased reactivity to novelty in the first year of life is generally maintained at later ages, including adolescence and adulthood, and correlates with increased anxiety and depression, as well as increased *amygdala* reactivity (Grogans et al., 2022; Grossmann, 2022; Roy et al., 2014; Fox et al., 2021). The alarm response to a stranger, as well as increased attention to faces showing fear (Grossmann and Jessen, 2017), has been identified as a component of child development in the second half of the first year of life (Sroufe, 1977). The fear reaction to the new, the unfamiliar, seems to be a characteristic of human primates in particular. It does not appear in other primates, except in more moderate forms (Herrmann et al., 2011), in which aversion or indifference to conspecific faces expressing fear is also visible (Kret et al., 2018). In the human species, therefore, the sight of a frightened face, as well as the scream or cry of a child, are social signals that communicate vulnerability and the need for help and encourage approach and the evolution of the human capacity for cooperation (Gračanin et al., 2018; Hammer and Marsh, 2015). Consequently, the *fearful ape hypothesis* (Grossmann, 2022) shows how living in small groups, characteristic of the social organization of the human species until the advent of agriculture, has led to a willingness to expect approach and help from a conspecific of the same group. This has qualified the protective withdrawal and the expression of fear or suffering of the *Despair* phase in a functional social signal of alert. This trait, as hypothesized by Grosman, which functiones in childhood and later in adulthood as a central element of the ability to cooperate in small groups, now persists in anxious and depressed individuals as a tendency to expect help or cooperation from others, in a social dimension that, especially since the Industrial Age, no longer functions in this relationship dynamic (Grossmann, 2022, 2023). These statements are not entirely shared by other scientists, who believe that the evidence is not strong enough to support these ideas (see Riddell et al., 2023). Grosmann's point of view on depression is particularly well suited to the anxious and depressive aspects of adolescence and young adulthood, when psychopathological manifestations are strongly activated by relational frustrations experienced as an inability to maintain a social bond and then as being outside the group. The depressive aspect, ostensibly a sign of renunciation, withdrawal and inability to achieve desired goals, appears in the *fearful ape hypothesis* as an expression of a childhood predisposition to exploit an expectation of cooperation that is no longer appropriate to the subject's current stage of development in a highly individualistic and competitive society (Humphrey et al., 2022). During human evolution, *Natural selection* (Vasseur and Quintana-Murci, 2013) has favored variants related to the *serotonin transporter gene (5-HTTLPR)*, with the *short allele* correlating with individuals an increased susceptibility to anxiety and depression in relation to stressful life events than individuals with the *long allele*, suggesting a gene-environment interaction (Caspi et al., 2003; Houwing et al., 2017; Wankerl et al., 2010). In animal models, maternal rearing appeared to “buffer” any potentially deleterious effects of the *short allele* on serotonin metabolism in monkeys who developed secure attachment relationships with their mothers during infancy (maternal “buffering”) (Kim et al., 2020; Meaney and Szyf, 2005; Suomi, 2011). Additionally, *short allele* carriers show increased *amygdala* response to unpleasant stimuli, thus indicating increased stress sensitivity (Munafò et al., 2008). From a social organization perspective, more collectivistic societies based on higher levels of cooperativism correlate with a higher number of individuals with *short allele* (Chiao and Blizinsky, 2010).

## Stress and the immune-inflammatory system

Vulnerability to depression appears to be associated with early exposure to stressful childhood experiences that have triggered *immune-inflammatory system* (Hamdani et al., 2012; Kozlowska et al., 2020; Rook et al., 2014). The latter, one of the earliest phylogenetically defense systems of organisms (Flajnik and Kasahara, 2009) and part of the composite stress system, is thought to have its own particular importance in the dynamics of depressive states (Hamdani et al., 2012). It represents a potential vulnerability factor also when fetal inflammatory events occur (Al-Haddad et al., 2019).

In close interaction with the integrated stress system has been the evolution of motivational/emotional systems and their behavioral and affective expression (Panksepp, 1998). Environmental and relational threats lead to the activation of the integrated system formed by the stress axis neurocircuit *Hypothalamic-Pituitary-Adrenal (HPA)*, the *autonomic system* (increased heart rate and blood pressure) and *inflammatory pathways in peripheral blood mononuclear cells*. This includes the activation of the *transcription factor nuclear factor-*κ*B* (NF-κB), increased circulating levels of *pro-inflammatory cytokines* such as *interleukin-6 (IL-6)* (Bierhaus et al., 2003; Miller and Raison, 2016; Raison and Miller, 2013). The inflammation theory of depression (Gałecki and Talarowska, 2018). is based on the vulnerability created in childhood by traumas or stressors that lead to an early activation of the *immune-inflammatory system* (Kozlowska et al., 2020; Miller and Raison, 2016). The activation or reactivation of this system would be directly linked to the possibility of becoming depressed (Beurel et al., 2020; Pace et al., 2006; Turkheimer et al., 2023).

The theory of inflammation in relation to depression shows that it is accompanied by behaviors such as slowing down, withdrawal, anhedonia. These behaviors are thought to have evolved in parallel with the inflammatory response because they are useful in the management of pathogen threats and implementing infection treatments (Raison and Miller, 2013). In preclinical models, pathological conditions elicit depression-like behavior, including apathy, anxiety, malaise, anorexia, insomnia and hyperalgesia (Stieglitz et al., 2015; Turkheimer et al., 2023). Pro-inflammatory cytokines induce a state of conservation-withdrawal. This is thought to be an evolutionary means of diverting metabolic resources to the costly tasks of immune activation, fever generation and tissue repair (Engel and Schmale, 1972; Raison and Miller, 2013). Inflammation can induce social isolation and withdrawal in humans, increasing neural sensitivity to social rejection, which in turn increases inflammatory responses to psychosocial stress (Eisenberger et al., 2010; Raison and Miller, 2013; Slavich and Irwin, 2014).

## Depression, social competition and social defeat

Social competition has been identified for many years as an important bio-psychosocial factor in the etiology of depressive functioning. It is linked to the experience of social defeat and so to the activation of submissive behavior (Gilbert, 1992; Gilbert et al., 2009; Price et al., 1994; Sloman et al., 2003; Sloman, 2002; Stevens and Price, 2000). The importance of this paradigm is that it has been extensively studied in animal models as a major source of stress in sexually mature individuals. Hence the analogy with responses to defeat in the human species, in both males and females, in terms of behavior, neurophysiology, neuroendocrine responses and depressive symptoms (see e.g., Hollis and Kabbaj, 2014; Scott and Fredericson, 1951; Kroes et al., 2007). Social competition is regulated by the Dominance system (Schjelderup-Ebbe, 1922; van der Westhuizen and Solms, 2015), already present in phylogenetically older vertebrates such as reptiles and fish, to regulate access to environmental food and sexual resources (MacLean, 1990; Toronchuk and Ellis, 2013). At present, neurobiological research does not recognize the presence of a specific primary emotional Dominance system, but may instead reflect an interaction of several basic, primary emotions (SEEKING, RAGE, FEAR and first experiences with the rough-and-tumble PLAY system), working together as a result of favorable outcomes during social development (Panksepp and Biven, 2012; van der Westhuizen and Solms, 2015). The *striatal system* and *brainstem* are the main sites involved in these functions (MacLean, 1990). Dominance and submissive behaviors are rooted in *serotonin* and *dopamine*-regulated neurocircuitry within the *basal ganglia* (Baxter, 2001, 2003; Toronchuk and Ellis, 2013). The evolution of the mammalian brain and then of the *limbic system* (*cingulate gyrus, parahippocampal gyrus, hippocampal formation, amygdala, septal area, hypothalamus*) (Rajmohan and Mohandas, 2007; Torrico and Abdijadid, 2023) has shaped the parallel evolution of Dominance dynamics. Indeed, the evolution of the limbic system has allowed the individual recognition of conspecifics within the mammalian group and has allowed the two contenders to share the same territory, without the looser having to flee, by the formation of rank structures. This reduces the repetition of conflictual behavior and distancing by establishing rank dynamics (Stevens and Price, 2000). The constitution of rank hierarchy has allowed the cohabitation of group of mammals, but it has not reduced the stress from competition and social defeat, but rather it has made it from being an acute event to one of potentially chronic stress (Sapolsky, 2004, 2005). In fact, the dynamics of rank hierarchy evolved through the behavior of submission or pacification, rather than distancing and fleeing by the loser, to defuse and inhibit the competitive aggression of the winner. The submission or pacification behaviors are species-specific and are characterized by an increased release of the *corticotropin-releasing factor (CRF)* (Kroes et al., 2006), decreased *serotonin* and *dopamine* (Johnson et al., 2012; Zhang et al., 2024) decreased *testosterone* (Sapolsky, 2004) and increased *interleukin-18* (Kroes et al., 2006). In addition, a review of three different neuroimaging studies supports the role of limbic, prefrontal and striatal pathways in the regulation of human rank dynamics (Beasley et al., 2012). Social defeat and the consequent submissive behavior generate stress (Deuter et al., 2021; Korzan and Summers, 2021; Sapolsky, 2005; Wohleb et al., 2011) and in humans, as already noted, these are considered to be analogous to depressive frameworks (Gilbert, 2000, 2006, 2021; Toronchuk and Ellis, 2013; Sloman and Gilbert, 2003; Kroes et al., 2007; Watt and Panksepp, 2009) and these influence epigenetic changes and transmission (Cunliffe, 2016).

The *neotenic* dimension contributed to making both the dynamics of Dominance and, therefore, of rank more stressful. The motivational/emotional systems that qualify a mammal, SEPARATION (PANIC/SADNESS), CARE, PLAY systems (Panksepp, 1998), with sexual development are powerfully connected to system, that of Dominance, whose basic behavioral patterns are already present in evolutionarily more archaic species such as reptiles (Baxter et al., 2001), fish (McGhee and Travis, 2010), crayfish (Issa et al., 2012; Panksepp and Huber, 2002), indicating that they originate from the ancient part of the brain that MacLean called the “reptilian brain” (MacLean, 1990). *Steroid hormones* are behavioral regulators and activators (de Vries and Södersten, 2009; Soares et al., 2010; Smiley et al., 2023). They have an important window of expression during puberty and adolescence. Puberty marks an important male/female distinction, the complexity of which was highlighted by the *Dual Hormone Hypothesis* (Mehta and Josephs, 2010). The latter suggests that the effect of *steroid hormones* on Dominance and submission behaviors are determined by the ratio of *testosterone* to *cortisol*. It shows that motivation for dominance correlates with high levels of *testosterone* and low levels of *cortisol*, whereas the opposite would occur in the experience of social defeat (Mehta and Josephs, 2010). In males, there is an exponential increase in the production of *gonadal testosterone* during adolescence, which drives competition. In females, the production of *androgenic hormones* is significantly lower than in males and is mainly of an *adrenal (suprarenal)* origin (Chronister et al., 2021; Cooke et al., 2020; Schulz and Sisk, 2016; Teo et al., 2023). This hormonal sex-specificity, which differentiates men from women, is in turn complicated by specific female sexual dynamics, with *progesterone* in particular increasing vulnerability to the experience of social defeat and depression (Forney et al., 2019).

As considered above, the *neotenic* aspect of the human species has favored the extension of the period in which the subject is in a “threatening” dimension that requires the presence and care of an adult for many years. At puberty, the significant increase in the drive for social competition within the paradigm of *Sexual selection* (Darwin, 1871) comes at an emotionally high price for mammals, particularly for the human species. The human *neotenic* dimension preserves the subject throughout life with characteristics that Lorenz (1971) describes as infantile. In other words, *neoteny* preserves the ability to play based on the motivations to be curious, to explore, to understand (Gilead, 2020). In addition to these characteristics, there remains in the human subject, after childhood, the need for relationships with other conspecifics. This leads to sexual-sentimental relationships (Romantic love), through which couple and family bonds are formed (Brumbaugh and Fraley, 2006), and the maintenance and creation of relationships within the group to which they belong. Both contexts highlight the active persistence of the need for social bonding in sexually mature individuals. The SEPARATION (PANIC/SADNESS) System is responsible for the wellbeing of feeling included and the discomfort of being excluded or rejected. One element that highlights the active presence of the SEPARATION (PANIC/GRIEF) system is the species-specific ability to cry, which appears very early in the child and continues until it is replaced by the production of visible tears. at the moment of separation from the caregiver (Bylsma et al., 2019; Gračanin et al., 2018). The ability to cry and produce visible tears persists throughout life as a signal of a state of fear, need for care or a signal of submission (Bylsma et al., 2019; Gračanin et al., 2018).

## Neoteny and learned-helplessness

The construction of personality has its central organizers in the primary motivational/emotional systems (Davis and Panksepp, 2018), which shape it through its endogenous endowment, influenced by both genetic and epigenetic components (Brown et al., 2020; Hochberg et al., 2011), in interaction with the experiential dimension. The latter is influenced by adaptation, modulated by learning, much of which is unconscious. One such unconscious learning is classical/Pavlovian conditioning, to which the learned helplessness paradigm can be traced (Seligman and Maier, 1967). The latter has highlighted how the mammalian brain, including the human species, when exposed to an uncontrollable experience, learns its inability to influence the outcome, resulting in a behavior of surrender, passivity, and consequently anxiety and depression (Maier and Seligman, 2016). This paradigm, first discovered as a behavioral modality (Seligman and Maier, 1967), was later complemented by a neuroscientific approach that revealed a specific neurotransmitter network and dynamics (Maier and Seligman, 2016, p. 32). These studies highlighted that initial experience with escapable shock “immunizes” whereas initial experience with inescapable shock result in the “learned helplessness” phenomenon (Maier and Seligman, 2016).

The learned helplessness paradigm again suggests the problem how much the depression-like in animal models is the same of human depression. Animal models are an invaluable resource for advancing our understanding of the pathophysiology of neuropsychiatric disorders and for developing new treatments and biomarkers (Nestler and Hyman, 2010; Sarnyai et al., 2011). Rodents, specifically mice, are popular in depression research due to their well-characterized genetics, ease of handling, and rapid reproduction. They permit the execution of controlled studies on the genetic, epigenetic, environmental, and pharmacological influences on depressive behaviors. For instance, the Chronic Mild Stress (CMS) model is a widely utilized rodent model for investigating depression (Willner, 2016). This model entails exposing animals to a series of mild, unpredictable stressors over an extended period. The objective of this model is to emulate the chronic stress that humans encounter, which has the potential to result in maladaptive alterations within the learning circuits, leading to the development of perseverative and inflexible coping strategies that contribute to the emergence of depressive symptoms (Cabib et al., 2020). Nevertheless, rodent models also exhibit several critical limitations. First, it is important to note that there are significant differences between human and animal brains regarding structure, function, and development. This complicates the process of translating findings from animal models to humans. For example, the period of adolescence in rodents is considerably shorter than that observed in humans, and the maturation of brain regions implicated in depression, such as the prefrontal cortex, does not align with the corresponding stages of human development (Chini and Hanganu-Opatz, 2020). Furthermore, the behaviors observed in animal models frequently represent simplistic approximations of the complex and nuanced symptoms observed in humans. In particular, rodent behaviors used to model depression (e.g., anhedonia, reduced activity) do not fully capture the complexity of human depressive symptoms (Gencturk and Unal, 2024). Moreover, while genetically modified animals help study specific gene functions, they may not capture the polygenic nature of most neuropsychiatric disorders. Furthermore, laboratory settings lack the complexity of human social and environmental contexts, which impacts the applicability of the results (see the Introduction for species-specific characteristics of rodents that show a relative lack of true separation distress; Panksepp and Biven, 2012, p. 321–322).

*Learned-helplessness* is a paradigm that can complement studies that have shown how prolonged neglect, repeated separations and highly insecure attachments can lead to depressive frameworks, as well as situations of prolonged social defeat, all situations in which the subject is exposed to the impossibility of modifying the stressful situation to which they are subjected.

The experiential field described above concerns the effects associated with a relational situation in which the individual's *BrainMind* (Panksepp, 1998) is urged to adapt/learn under the impetus of relational environmental inputs. In addition to this experiential dimension, it has been increasingly emphasized that mere exposure to the behavior of a conspecific determines mimesis activity and thus learning in the individual, as we will see in the next section.

## Observational learning and identification

Unlike classical/Pavlovian conditioning, in which learning occurs only through direct experience, there is another type of learning that occurs through the process of watching others and then imitating what they do, called observational learning. *Neoteny* has increased the importance of early learning for our species (Tottenham, 2020), learning that is largely implicit. An individual, particularly from infancy to adolescence, shapes their own behavior and emotional responses largely on the basis of exposure to the behavior and emotional expressions of a significant other. The observation of parents is central in this respect, and this qualifies a particular model of learning, called, as mentioned above, *observational learning*, by Bowlby (1969) in his studies on Attachment. *Observational learning* was first studied by social cognitivism (Bandura, 1986; Fryling et al., 2011), which emphasized its function in shaping the individual 's personality and behavior as a result of their exposure to the behavior or regulation of parental motivational/emotional systems (Bandura, 1986; Hunnius and Bekkering, 2014). The subject is therefore exposed to a “heteroregulation” brought about by the implicit learning of regulatory models of the motivational/emotional systems, modeling a response without the individual first associating it with a stimulus-response experience. Evidence of an innate predisposition to imitation in the human species was provided by Meltzoff and Moore's (1977) now historic studies of imitation in infants. Based on repeated studies, Meltzoff (2005) developed the “*like me”* hypothesis, based on the child's predisposition to imitation as a means of experiencing the mental state associated with the behavior being imitated, and consequently the ability to understand other minds.

The discovery of *mirror neurons* (Gallese et al., 1996; Rizzolatti et al., 1996), provided a neuro-anatomical and neuro-functional basis for the paradigm of *observational learning*. It has been shown that these populations of neurons determine the function of *embodied simulation* (Gallese, 2009), namely, the actions seen and inferred in their intentionality activate similar *premotor cortex area* in the observer (Coudé et al., 2016; Gallese et al., 2009; Iacoboni, 2005). From birth, children witness the world unfolding around them, and their *mirror systems*, through *embodied simulation*, expose them to a vicarious experience which helps to calibrate emotional systems, leading to implicit learning that at the same time contributes to the structuring of personality (Gallese, 2009). However, from the discovery of mirror neurons to the present day (Bonini et al., 2022), evidence has accumulated that simulation is a highly integrated process that seems to involve a mosaic of affective, motor and somatosensory components (Bastiaansen et al., 2009), highlighting the need to understand the “social brain” not in terms of specific structures, but rather in terms of their interaction in networks (Kennedy and Adolphs, 2013).

Thus how much and how *mirror systems* contribute to social learning in relation to the developmental and structuring role of identifications as tools for personality formation is still much debated (Del Giudice et al., 2009; Ferrari et al., 2017; Heyes and Catmur, 2022; Keysers and Gazzola, 2014; Simpson et al., 2014).

Regarding transgenerational transmission the paradigm of the contagion effect within family interactions is of particular interest to understand depression (Paz et al., 2021; Prochazkova and Kret, 2017; Salazar Kämpf and Kanske, 2023).

The transgenerational dynamics that contribute to personality formation and the vulnerability or resilience of psychopathological dimensions such as depression, articulate with another important vector, *epigenetics* (Brown et al., 2020; Ferrari et al., 2013; Hofer, 2014), through which the regulation of neurofunctional systems are passed from one generation to the next (Monaco, 2021; Schiele et al., 2020).

## Depression as an inhibition of the SEEKING disposition

Of all the emotions described by Panksepp, the SEEKING disposition has been identified as the most important and the oldest from a phylogenetic point of view (Panksepp, 1998). It is an instinctual function of the mind/brain that drives organisms to explore the environment and to seek and approach every type of resource necessary for survival and reproduction (Ikemoto and Panksepp, 1990; Alcaro et al., 2007). Studies of the neurobiological processes involved in SEEKING behaviors converge with the dominant role played by the *mesolimbic dopaminergic system* (ML-DA) and its associated areas and circuits (Alcaro and Panksepp, 2011).

The SEEKING disposition constitutes the basic emotion of all motivated behaviors and, in particular, of the appetitive phase, the inclination to search for a distal stimulus, which is usually distinguished from the consummatory phase, the consumption of a proximal stimulus (Wise and Bozarth, 1987; Berridge and Robinson, 1998; Salamone and Correa, 2012). Its activation is proportional to the motor, cognitive and emotional efforts that the organism can recruit to achieve its objectives (Salamone and Correa, 2012).

Although it has traditionally been associated with movements of exploration and approach, the SEEKING disposition is also activated in environmental conditions characterized by the presence of dangers, threats, frustrations or other forms of impediment. In these cases, it supports energetic behaviors aimed at seeking safer and less frustrating conditions (Ikemoto and Panksepp, 1990; Alcaro et al., 2007) and is also recruited in various manifestations of predation and approach toward competitive behaviors (search for prey, approach toward an enemy, etc.) (Giacolini and Sabatello, 2019; Giacolini et al., 2021).

The SEEKING disposition is maximally activated in conditions of novelty or uncertainty, while it is much less involved in situations where environmental conditions are predictable and the behaviors elicited are already pre-established (Watabe-Uchida et al., 2017; Schultz et al., 2017). From a cognitive point of view, SEEKING is a fundamental vector of all non-automatic functions linked to an expectation of the outcomes of actions, and a necessary component of decision making (Westbrook et al., 2020). Furthermore, its activation signals an “error” in the organism's system of expectations, when a stimulus with appetitive or incentive qualities unexpectedly appears in the organism's perceptive field (Schultz, 2017, 2019). In this sense, it plays an essential function in all forms of appetitive learning based on the presence or anticipation of rewards.

From an affective point of view, the SEEKING disposition manifests itself as sensations of enthusiasm, desire, interest, curiosity and optimism (Alcaro and Panksepp, 2011). Therefore, from a neuro-psychoanalytic perspective, the SEEKING disposition has been considered the emotional substrate of “libido,” implying not only sexual energy, but a source of direct engagement with the external world (Kaplan-Solms and Solms, 2000). Although it can be directed toward a specific goal, which coincides with its achievement, it has a margin of functional autonomy and can be activated independently of predetermined motivations or goals. As such, it acts as a non-specific drive toward exploration and contact. It is responsible for an openness toward novelty and a motivation toward contact with the “Other-than-Self,” which constitute the essential conditions for organic individuality to emerge from rigid sensorimotor automatism. It opens the way to the emergence of a subjective phenomenological field intrinsically linked to it (Alcaro, 2019, 2021).

Various experimental and clinical data from animal models and human patients indicate that blocking the SEEKING function is thought to results in a depressive withdrawal that isolates the subject from the world and from any form of contact with the Other-than-Self. For example, in laboratory animals, large brain lesions of the SEEKING system result in a drastic reduction in spontaneous activity and without forced feeding these animals will allow themselves to starve to death. More moderate lesions or chemical inhibition of the SEEKING system can instead block approach and reward-seeking behaviors and a decrease in behaviors engaged in escaping stressful environmental conditions (see Alcaro and Panksepp, 2011).

Moreover, neurological patients who have suffered brain lesions in regions of the SEEKING circuit show a clear decrease in motivation and depressive tendencies (Farinelli et al., 2013). Therefore, animal and human data indicate that a neuroanatomical deficit affecting the SEEKING system results in a form of depressive withdrawal which may be manifested in two main ways: anhedonia, i.e., the loss of enthusiasm and interest in the world, and learned helplessness—an implicit assumption of the inevitability of adverse conditions or events (Shumake and Gonzalez-Lima, 2003; Di Chiara et al., 1999; Nestler and Carlezon, 2006; Alcaro and Panksepp, 2011).

It has been largely demonstrated that uncontrollable aversive experiences produce neurochemical and structural modifications within brain structures of the limbic system, such as the amygdala, the hippocampus and the prefrontal cortex that appear to be responsible for an inhibition of the response of some subcortical systems. This inhibition results in a form of surrender (learned helplessness), a renunciation of any attempt to actively seek a means of escape from the adverse condition (Alcaro et al., 2007; Alcaro and Panksepp, 2011).

Although research related to the learned helplessness model has highlighted the predominant role of serotonergic transmission, numerous studies have also indicated a consistent reduction in the activity of the *mesolimbic dopaminergic system* (ML-DA), the main ascending pathway of the brain's SEEKING system. These neurochemical and behavioral effects are also counteracted by the prolonged administration of antidepressant treatments, whose effectiveness in modifying the condition of learned helplessness seems directly linked to their ability to restore normal functioning of the *mesolimbic dopaminergic system* (Alcaro and Panksepp, 2011).

Repeated or chronic exposure to unavoidable stress not only results in a diminished ability to actively avoid or fight, but can also lead to a reduction of interest in all stimuli and activities that normally have a rewarding value (anhedonia^1^) (Scheggi et al., 2018). This anhedonic condition is directly linked to a reduced functionality of the SEEKING system, and in particular of the DA ML circuit, which, as we have already underlined, constitutes the neurobiological substrate that mediates a state of incentive activation necessary to perceive and explore the qualities of rewarding stimuli and to pursue approach behaviors (Alcaro and Panksepp, 2011; Russo and Nestler, 2013).

The hypothesis that a deficit in the SEEKING system plays a determining role in depression has been confirmed by studies of human patients who show a reduction in the activity of the *mesolimbic dopaminergic system* or other neuronal sites belonging to this emotional system (Mayberg et al., 2000; Alcaro and Panksepp, 2011; Salone, 2021). This hypothesis has also recently led to the development of new antidepressant treatments based on the direct stimulation of brain areas or circuits that belong to the SEEKING system (Coenen et al., 2010; Döbrössy et al., 2015; Schlaepfer et al., 2013, 2014). This research has also indicated that the antidepressant effectiveness of electrical stimulation is linked to the restoration of normal functioning of the DA ML system (Thiele et al., 2020).

In conclusion, therefore, the neuro-ethological research conducted in recent decades on animals and humans has made it possible to qualify depression as a pathological condition linked to a psychobiological state characterized by a diminished functionality of the SEEKING disposition. Seen in this light, the main symptoms of depression, namely anhedonia, impotence and loss of hope, can be traced back to a single emotional deficit: a defect in the ability to spontaneously generate states of confident expectation and appetitive motivation.

## Depression in adolescence and young adulthood

Human personality is rooted in the primary emotional systems (Davis and Panksepp, 2018), while character traits are shaped through the gradual secondary learning processes (Montag and Panksepp, 2017). These processes take place in childhood, mainly through family relationships (Stern, 1985), and stabilize in adolescence and early adulthood, when sexual maturation contributes significantly to pushing the individual out into the relational world outside the family (Backes and Bonnie, 2019, p. 37–76). Imitative or identification processes are modes of learning that have been selected because they are functional and promote adaptation to an environment that tends to be predictable and stable (Maranesi et al., 2014; Rizzolatti et al., 2014). This predictability has probably been made less effective by changes in contemporary Western societies (Blokker and Vieten, 2022; Crane-Kramer and Buckberry, 2023; Crouch et al., 2019; Haugan, 2023; Hidaka, 2012). Closely related to the above is the increase in the individualistic and competitive dimension that has characterized Western societies in recent decades, and which appears to have had a massive impact on adolescents and young adults (Kafetsios and Sideridis, 2006; McLoughlin et al., 2022; Smith et al., 2011). These dimensions, which characterize the development of Western societies, show a higher level of wellbeing when compared to collectivist cultures. However, this comparison tends to become negative when one analyses the wellbeing of a single individual (Humphrey and Bliuc, 2022). Freedom and autonomy, characteristics of highly individualistic Western societies, are increasingly showing the dark side of mental health-related malaise, particularly among adolescents and young adults (Humphrey et al., 2022), along with an impoverishment of mental life (Botha et al., 2023), the latter to be considered in correlation with the development of the Internet (Prasad et al., 2023). This development over the last two decades, with all its articulations, and the increase in the technology of devices, both computers and especially smartphones, have constituted an alarming source of mimesis. This has changed the processes of transgenerational transmission, which was mainly based on interaction with family members. The development of exposure to behavioral patterns conveyed by social media (Liu et al., 2016; Stangl et al., 2023) and electronic devices has made the “matching problem” of imitative processes, specifically, the problem of matching observed actions with their imitation and transposition into behavioral expressions of motivational/emotional systems has become particularly dramatic (Brass and Heyes, 2005). This dimension is articulated by a progressive tendency, among adolescents and young adults, to spend more time on social media and less time in the presence of other peers, this correlates with a reported lower wellbeing and greater susceptibility to depression (Lin et al., 2016; Shakya and Christakis, 2017; Twenge, 2020; Twenge et al., 2022; Wadsley and Ihssen, 2023; Weinstein, 2023).

The transformation of the cultural context, and so of the relational modalities considered above, is combined with the species-specific aspects of neurophysiological and sexual development already considered in previous sections, to which some further aspects must be added. The psychobiological development of an adolescent continues into young adulthood. However, it is characterized by more continuity than the discontinuity that characterized the transition from childhood to adolescence. Particularly during young adulthood, the development of the connectivity of the *cortico-basal ganglia-thalamo-cortical* loop network involved in the functions of motivation and emotion processing continues (Haber and Calzavara, 2009; Raznahan et al., 2014) and, specifically, the *cortico-striatal-thalamic-cortical* loop connectivity in relation to the connections with the *orbitofrontal area of the prefrontal cortex* (Fettes et al., 2017). The development of this network is paralleled by the development of its components, not only of the *cortical* but also of the *subcortical areas* (Raznahan et al., 2014). These maturational processes, which continue into young adulthood, are characterized by sexually dimorphic *subcortical* maturation, with males reaching peak volume in *striatal* and *thalamus* areas later than females, a *heterochrony* related to earlier advent puberty in females that in contrast to the greater *neoteny* in males (Petanjek et al., 2011). This maturational dimorphism interacts with the dynamics of sexual development, favoring affective disorders in females and behavioral disorders in males (Rice et al., 2018; Rubinow and Schmidt, 2019). In both sexes, peak maturation of the *striatum* and *thalamus* occurs after that of the *cortex* (Raznahan et al., 2014). This may indicate a dimension of the exploratory drive and the ability to experience pleasure and learning that is still developing in both sexes. At the same time, *synaptic pruning* and *myelination* continue (Petanjek et al., 2011), which, together with a gradual reduction in *gray matter* thickness in the *prefrontal cortex*, favors the development of specialization in both cognitive and motivational functions (Spear, 2013). In young adulthood, the development of *white matter fibers* between the *amygdala* and *prefrontal cortex* continues, increasing the modulation of the former (Goetschius et al., 2019; Paus et al., 2001). Pubertal development, which is characterized by hormonal changes, has a major influence on the differentiation of brain functions in the two sexes (Raznahan et al., 2014). Both are involved, as stated above, in the integration of the two motivational/emotional systems, namely the SEPARATION (PANIC/GRIEF) system and the Dominance systems. This process, which begins in adolescence and continues into young adulthood, will have gender-specific characteristics. In women, as has been mentioned before, the production of less testosterone makes them more vulnerable to confrontational and competitive stress, with a higher incidence of the *cortisol* effect (Zajkowska et al., 2022). This is combined with the effect of *progesterone* produced during the *luteal phase*. *Progesterone* increases connectivity between the *amygdal*a and the *Default Mode Network* (DMN) (Beltz and Moser, 2020; Kiesner et al., 2020), which favors the memory negative events and, therefore, the depressive dimension (Andreano et al., 2018).

## Conclusion

This work takes depression to be an adaptive response that has evolved in the phylogeny of the human species, that reflects the combined interactions of multiple neurobiological systems in a complex, multi-level recursion (Dean and Keshavan, 2017; Watt, 2023).

Depression, more specifically, is first and foremost an expression of the stress of separation and loss, as well as a functional expression of an evolutionarily conserved mechanism to terminate separation distress (Watt and Panksepp, 2009; Watt, 2023). The *BrainMind* development (Panksepp, 2010) of the human species have been made possible by *neoteny* that slows growth and development, that extends the period of the earliest social bond to the mother and parental figures until puberty and also after with Romantic love and the need for social bonds (Silk, 2014). At the same time from puberty onwards, under the pressure of hormonal and sexual maturation, an individual is exposed to the effects of stress caused by social competition far more than in childhood. If separation anxiety is reduced and silenced by an adaptive response that has evolved in the phylogeny of human species (Watt and Panksepp, 2009; Watt, 2023), social anxiety can be seen as a propensity to act submissively to reduce relational conflict and avoid negative consequences and rejection by others (Dijk et al., 2018). This can lead to a depressive state as the ultimate expression of submissive behavior (Price, 2002; Price and Gardner, 2004). Although depression and anxiety are traditionally conceptualized as two independent disorders, they are highly co-morbid, with rates ranging from 40 to 50% (Ter Meulen et al., 2021). In particular, social anxiety is a comorbidity most commonly found among patients with depressive disorders (Ionescu et al., 2013; Stein et al., 2001), constituting an aggravating factor of the former (Ter Meulen et al., 2021), with increased risk of recurrence and reduced functionality (Koyuncu et al., 2019). Social anxiety tends to occur in adolescence frequently anticipating depression, particularly in adolescents and young adults (Koyuncu et al., 2019) and is characterized, particularly in males, by a reduction in testosterone (Maner et al., 2008), which is believed to be central to the dominance system (Van der Westhuizen, 2013). Therefore, depression can be considered as a consequence of separation anxiety and of social anxiety within the dynamics of social competition. Watt writes: “Notably, Nesse (2000) hypothesized that depression might also function adaptively to terminate dominance conflicts that pose a survival threat if undesirably protracted. We hypothesized that selection effects would be amplified by a functional utility of the same mechanism at both early, highly vulnerable ages of infancy, and then later in adolescent and maturing animals pursuing dominance and its conjoined procreative opportunities and risk of serious injury” (Watt, 2023, p. 5). And Nesse (2000, p. 17) writes:” Just as anxiety inhibits dangerous actions, depression inhibits futile efforts.” Depression, therefore, seems to have two possible causes, one related to separation anxiety and the other to the anxiety of failure in the social competition for dominance. This subdivision can be compared to the one proposed by S. Blatt in the field of psychopathology. He proposes a distinction between anaclitic depression, characterized by a preoccupation with abandonment and the loss of interpersonal relationships, and introjective depression, linked to the drive for competition and accompanied by a sense of failure and self-criticism (Blatt et al., 1982; Blatt and Luyten, 2009). But Watt warns: “One basic symptomatic bifurcation seems to rest between agitation (suggesting an anxious-depression phenotype) and psychomotor retardation (suggesting a more melancholic or apathetic phenotype). Despite this being one of the most long term phenotypic distinctions made in depression, going back to earlier versions of DSM, there is still no unambiguous biological evidence” (Watt, 2023, p. 8).

Both depressions are characterized by a mechanism of anxiety reduction, separation anxiety in one and social anxiety related to the relational threat of dominance in the other. Depression associated primarily with the SEPARATION (PANIC/GRIEF) system has as its central element a reduction in the activity of the SEEKING system, whereas depression associated with the dynamics of social competition and dominance has as its central element a reduction in testosterone production (Määttänen et al., 2021), the administration of which has been shown to have an antidepressant function (Anderson et al., 2022). Finally, it should be remembered that the neurobiology of depression is significantly influenced by the immune-inflammatory system, which is conjoined with the stress axis HPA. Pro-inflammatory cytokines disrupt negative feedback from cortisol on the HPA axis, preventing normalization/down regulation of CRF (Watt, 2023, p. 21) as well as reducing the serotoninergic and dopaminergic systems (Watt, 2023, p. 24; for an exhaustive review of these complex, multilevel recursions see Watt, 2023).

The work of integrating the SEPARATION (PANIC/GRIEF) system with social competition and the dynamics of Dominance which particularly engages adolescents and young adults, is closely intertwined with the environmental and relational contexts that shape the character traits embedded in the primary motivational/emotional systems mentioned above (Montag and Panksepp, 2017). Relational contexts, especially those related to parent-offspring relationships, have the power to shape the latter's character, mainly through the processes of identification mediated by *observational learning* and embodied simulation (Gallese, 2009) as described in this article, through which the transgenerational transmission that is so prevalent in depression is enacted (Ferrari et al., 2013; Goodman, 2020).

The bio-psycho-social characteristics of adolescence and young adulthood described above are articulated with the specificity of the human *BrainMind* (Panksepp, 2010) and its vulnerability or capacity to experience depression and anxiety. The evolution of the human brain and in particular of the *cerebral cortex*, has always led an individual to an awareness of the future and a memory of the past (Edelman and Tononi, 2000). This awareness can itself contribute to a predisposition to a particular form of depression, called *Existential depression* (Berra, 2021). This depressive aspect, described as *non-pathological* (Berra, 2021), is related to the human species-specific *cortical* motivation to seek meaning and understanding of phenomena driven by the SEEKING *subcortical* system (Panksepp, 1998), which drives all vertebrates to explore their environment. The *corticalisation* of the exploratory drive (Davis and Panksepp, 2018) gives form to the motivation to seek meaning and understanding of an individual's presence in the world, exposing them to the despairing experience of finding meaning in existence (Pellens et al., 2022). This cognitive experience may determine a reduction in humans to *vitality affect*s (Ammaniti and Ferrari, 2013; Stern, 2010), exposing and drawing them to desperate nihilism (Wang, 2022), particularly present in adolescence and young adulthood.

Depression, therefore, reveals a complexity of causes and manifestations for which Watt's indications are useful: “None of these ideas about the putative selection mechanism(s) in relationship to depressive process should be seen as necessarily competitive or mutually exclusive in the least” (Watt, 2023, p. 6).

Finally, the limitations of this work are mainly to be found in absence of a Cross-Cultural Perspective. The paper primarily focuses on young adults in Western societies. This being said, the authors are aware that adolescents and young adults from different cultural backgrounds around the world may have different experiences. Moreover, this aspect is increasingly articulated with both the phenomenon of the proliferation of devices and Internet in all non-Western cultures (Lopez-Fernandez et al., 2023; Roy and Das, 2022) and with migration, both have implications for transcultural psychiatry and ethnopsychiatry (Caroppo et al., 2009, 2023; Osborn et al., 2021). How these phenomena influence the regulation and psychopathological expression of primary emotional systems in adolescents and young adults born in non-Western societies may be an interesting area for future research.

## Footnotes

^1^Defined in the DSM V as “the decreased ability to experience pleasure from positive stimuli or a degradation in the recollection of pleasure previously experienced,” anhedonia is considered among the main symptoms for the diagnosis of major depressive disorder in men (DSM-5, American Psychiatric Association, 2013).
